# Physiological, epigenetic and genetic regulation in some olive cultivars under salt stress

**DOI:** 10.1038/s41598-018-37496-5

**Published:** 2019-01-31

**Authors:** Soraya Mousavi, Luca Regni, Marika Bocchini, Roberto Mariotti, Nicolò G. M. Cultrera, Stefano Mancuso, Jalaladdin Googlani, Mohammad Reza Chakerolhosseini, Consolación Guerrero, Emidio Albertini, Luciana Baldoni, Primo Proietti

**Affiliations:** 1 Università degli Studi di Perugia, Dept. Agricultural, Food and Environmental Sciences, Perugia, Italy; 2 CNR - Institute of Biosciences and Bioresources, Perugia, Italy; 3 Università degli Studi di Firenze, Dept. Agrifood Production and Environmental Sciences, Florence, Italy; 4 Ferdowsi University of Mashhad, Dept. Soil Science, Mashhad, Iran; 5 Universidad de Malaga, Dept. Molecular Biology and Biochemistry, Malaga, Spain; 6 Present Address: CNR - Institute of Biosciences and Bioresources, Perugia, Italy

## Abstract

Cultivated olive, a typical fruit crop species of the semi-arid regions, could successfully face the new scenarios driven by the climate change through the selection of tolerant varieties to salt and drought stresses. In the present work, multidisciplinary approaches, including physiological, epigenetic and genetic studies, have been applied to clarify the salt tolerance mechanisms in olive. Four varieties (Koroneiki, Royal de Cazorla, Arbequina and Picual) and a related form (*O*. *europaea* subsp. *cuspidata*) were grown in a hydroponic system under different salt concentrations from zero to 200 mM. In order to verify the plant response under salt stress, photosynthesis, gas exchange and relative water content were measured at different time points, whereas chlorophyll and leaf concentration of Na^+^, K^+^ and Ca^2+^ ions, were quantified at 43 and 60 days after treatment, when stress symptoms became prominent. Methylation sensitive amplification polymorphism (MSAP) technique was used to assess the effects of salt stress on plant DNA methylation. Several fragments resulted differentially methylated among genotypes, treatments and time points. Real time quantitative PCR (RT-qPCR) analysis revealed significant expression changes related to plant response to salinity. Four genes (*OePIP1*.*1*, *OePetD*, *OePI4Kg4* and *OeXyla*) were identified, as well as multiple retrotransposon elements usually targeted by methylation under stress conditions.

## Introduction

Cultivated olive (*Olea europaea* subsp. *europaea* var. *europaea*) is a long-living, evergreen, thermophilic species. Due to erratic global climate change, plant crops are frequently exposed to several abiotic stresses as salinity, causing productivity reduction and vegetative decline^1,2^. Although irrigated areas will increase for olive cultivation in the near future, water scarcity, in the Mediterranean basin and in the new countries where cultivation is expanding, will limit or restrict the use of fresh water in agriculture, especially in most coastal areas, where olive is mainly cultivated. Furthermore, salinity is becoming a major problem due to the high rates of evaporation, insufficient leaching and a large quantity of saline water used for olive irrigation^3^.

Plant tolerance to salinity stress involves the activation of physiological complexes, metabolic pathways and molecular networks. A deeper understanding on how plants respond to different level of salinity stress and the development of integrated combination of genetic, epigenetic and physiological techniques will represent important pre-requisites for the development of salt-tolerant varieties^4^.

Salinity stress affects fundamental processes of cellular function, including cell division, differentiation and expansion, which play substantial impact on plant growth and development^5–9^. Long-term exposure to salinity induces ionic stress, which leads to premature senescence of adult leaves, and thus to reduced rates of photosynthesis^10,11^. Recent research has identified various adaptive responses to salinity stress at molecular, metabolic and physiological level, although mechanisms underlying salinity tolerance are far from being completely explained.

In olive trees salt tolerance is related to mechanisms preventing salt translocation or decreasing its transport, excluding Na^+^ and Cl^−^ from leaves^12–14^, or compartmentalizing toxic ions within leaves^15,16^.

Understanding the epigenetic regulation of plant response to abiotic stresses could help unraveling genetic variation for improving crop productivity as well as adaptation to stress conditions^17^. Analysis of candidate genes and studying their regulation in response to abiotic stresses represent common ways to understand plant adaptation ability^18^. When plants are exposed to stressful conditions, including salinity, the mechanisms of adaptation are activated, allowing the plant to react. In this regard, cytosine methylation may play an integral role in the regulation of gene expression at both, transcriptional and post-transcriptional levels^19,20^. DNA methylation is very sensitive to different stresses and indicates how plants adapt themselves to cope with environmental constrains. Several studies have demonstrated that cytosine methylation plays an important role in regulating various biotic and abiotic stresses, such as low temperature^21–23^, water deficiency^24^, bacteria blight^25^, ion implantation^26^, heavy metals^27^, low nutrients^28,29^, tissue culture^30,31^ and salt stress^32^.

Genes induced/repressed by salt stress have been studied in numerous plant species, such as Arabidopsis^33^, poplar^34^, rice^18^ and soybean^35^. Several genes are associated with the stabilization of photosynthesis^36^, the improvement of ion distribution and the maintenance of osmotic balance^37^. The coordination of mannitol transport with intracellular metabolism^38^, reduction of membrane structures, enhancement of osmotic adjustments and antioxidative enzyme regulation under salt stress^39^ were also reported. Moreover, the transcriptome profile was explored and differentially expressed genes related to salt tolerance were identified in olive^9,40^, as well as in other crop species^36,41–46^.

The aim of this work was to characterize the genetic and epigenetic response to salt stress in physiologically monitored olive plants. Experiments were carried out on plants of different genotypes in a hydroponic system in order to control plant nutrient uptake and to better monitor the physiological and epigenetic changes in response to salinity. Differentially methylated genes were fully characterized and their expression was evaluated among cultivars under different stress levels.

## Results

### Plant growth and visible symptoms

**Figure 1:**
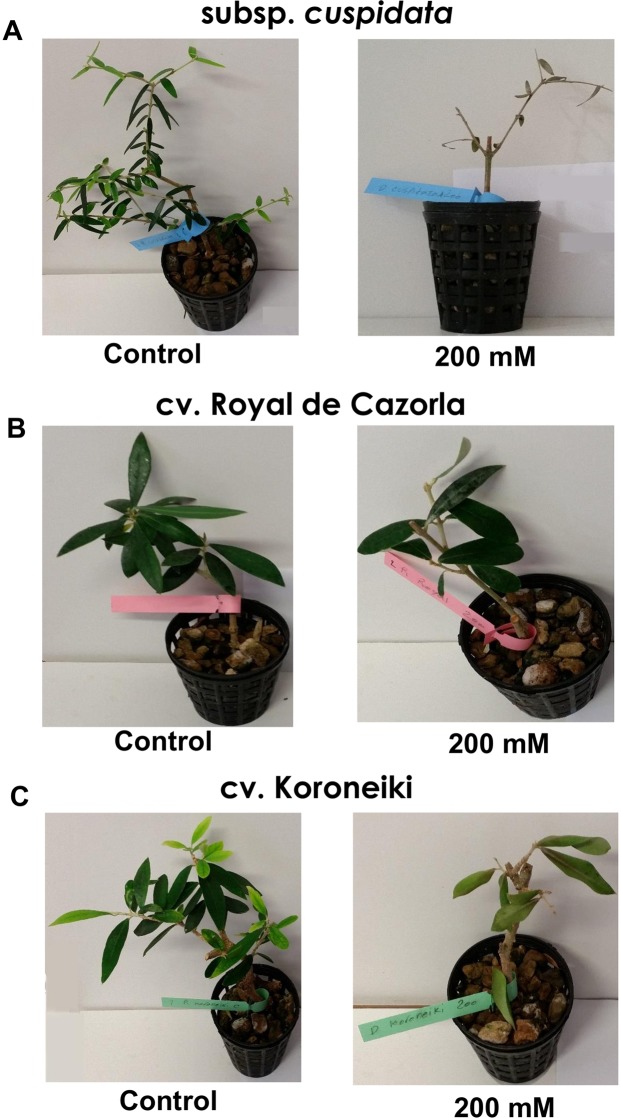
The plants growing condition in control and 200 mM of NaCl treatment. (**A**) Plants of subspecies *cuspidata* (after 14 DAT), (**B**) Plants of cv. Royal and (**C**) Plants of cv. Koroneiki (both after 43 DAT).

Among the five studied genotypes, the *cuspidata* samples resulted extremely susceptible to NaCl treatment and all replicates died after 14 days since treatment starting, while the control plants were growing well in hydroponic condition, therefore it was not possible to include them in further experiments (Fig. 1A). About the four analyzed cultivars, the symptoms of salt stress in Arbequina and Picual were intermediate (Table 1), therefore, the physiological and molecular analyses were performed only on the most tolerant (Royal) and most susceptible (Koroneiki) cultivars.

**Table 1:** Plant growth measurement of four cultivars at 0-100-200 mM NaCl, after 60 DAT of hydroponic culture.

Cultivar	NaCl (mM)	L DW (g)	Sh DW (g)	St DW (g)	R DW (g)	Above/below ratio	Plant h (cm)	n. Lat sh	Len lat sh (cm)	n. L	L Area (cm^2^)
Royal	0	0.42^a*^	0.06^a^	3.83^a^	1.20^a^	3.59^a^	16.0^a^	2.40^a^	8.9^a^	12.0^a^	100^a^
	100	0.31^ab^	0.05^a^	2.51^b^	0.92^b^	3.12^a^	16.0^a^	2.01^a^	6.5^ab^	8.00^b^	67.2^b^
	200	0.18^b^	0.08^a^	1.97^c^	1.38^a^	1.62^b^	15.4^a^	1.30^b^	4.6^b^	7.00^b^	58.8^b^
Koroneiki	0	4.57^a^	1.24^a^	7.27^a^	7.21^a^	1.81^a^	15.0^a^	3.10^a^	18^a^	30.0^a^	180^a^
	100	1.92^b^	0.40^b^	3.70^b^	3.89^b^	1.55^a^	15.2^a^	1.80^b^	5.5^b^	13.0^b^	72.8^b^
	200	1.42^b^	0.11^b^	1.70^c^	2.38^b^	1.36^a^	13.0^a^	1.75^b^	3.2^b^	9.4^c^	52.6^c^
Picual	0	1.23^a^	0.53^a^	1.71^a^	2.93^a^	1.18^a^	16.5^a^	3.60^a^	27.0^a^	14.0^b^	84.0^a^
	100	1.51^a^	0.27^b^	1.15^ab^	2.09^a^	1.40^a^	14.3^ab^	1.80^b^	13.4^ab^	16.0^a^	90.0^a^
	200	1.03^b^	0.29^b^	0.97^b^	1.73^b^	1.32^a^	11.8^b^	1.10^b^	11.2^b^	11.4^c^	64.4^b^
Arbequina	0	1.03^a^	0.28^a^	2.23^a^	3.01^a^	1.18^a^	18.7^a^	4.50^a^	17.2^a^	14.0^a^	56.0^a^
	100	0.45^b^	0.39^a^	1.20^ab^	1.97^b^	1.04^a^	16.2^a^	2.10^ab^	13.3^b^	8.00^b^	32.0^b^
	200	0.32^b^	0.25^a^	0.78^b^	1.35^b^	1.00^a^	12.2^b^	1.50^b^	11.2^b^	7.00^b^	28.0^b^

The abbreviated data are: NaCl concentration, leaves dry weight, shoots dry weight, stem dry weight, root dry weight, above ground/below ground ratio, plant height, number of lateral shoots, length of lateral shoots, number of leaves, leaf area. Data represent the average value per plant (three replicates per variety).

Means followed by different uppercase letters are significantly different at P ≤ 0.05.

The symptoms appeared 30 Days After Treatment (DAT) in ‘Koroneiki’ and ten days later in ‘Royal’ and became severe at the end of the experiment. At 100 mM NaCl, the cv. Koroneiki did not show clear symptoms related to saline stress and plants were able to produce some new leaves. In cv. Royal, even though the stress symptoms were not present, apical leaves did not grow properly. At 200 mM NaCl, the cv. Koroneiki was strongly stressed and leaves dried suddenly, while in cv. Royal any symptom was present, neither growth of apical leaves (Fig. 1B,C).

At 60 DAT, dry weight (DW) decreased dramatically in ‘Koroneiki’ leaves compared to the control (57.9% and 68.9% at 100 and 200 mM NaCl, respectively), whereas ‘Royal’ showed significant reductions of leaf DW only at 200 mM NaCl (57.1%), also in ‘Picual’ and ‘Arbequina’, leaf DW strongly decreased in stressed plants (Table 1, Fig. S1). Root DW remained constant in cv. Royal, but in cv. Koroneiki it decreased 46.0% and 67.0% at 100 and 200 mM NaCl, respectively. The stem weight was reduced significantly in ‘Royal’ treated at 200 mM NaCl (48.56%), while no differences were observed for shoot DW. The reductions of shoot and stem DW at 200 mM in ‘Koroneiki’ were 91.1% and 76.6%, respectively. Also, in ‘Picual’ and ‘Arbequina’, root and stem DW decreased in stressed plants, except at 100 mM in ‘Picual’.

### Gas exchange, chlorophyll and relative water content

**Figure 2:**
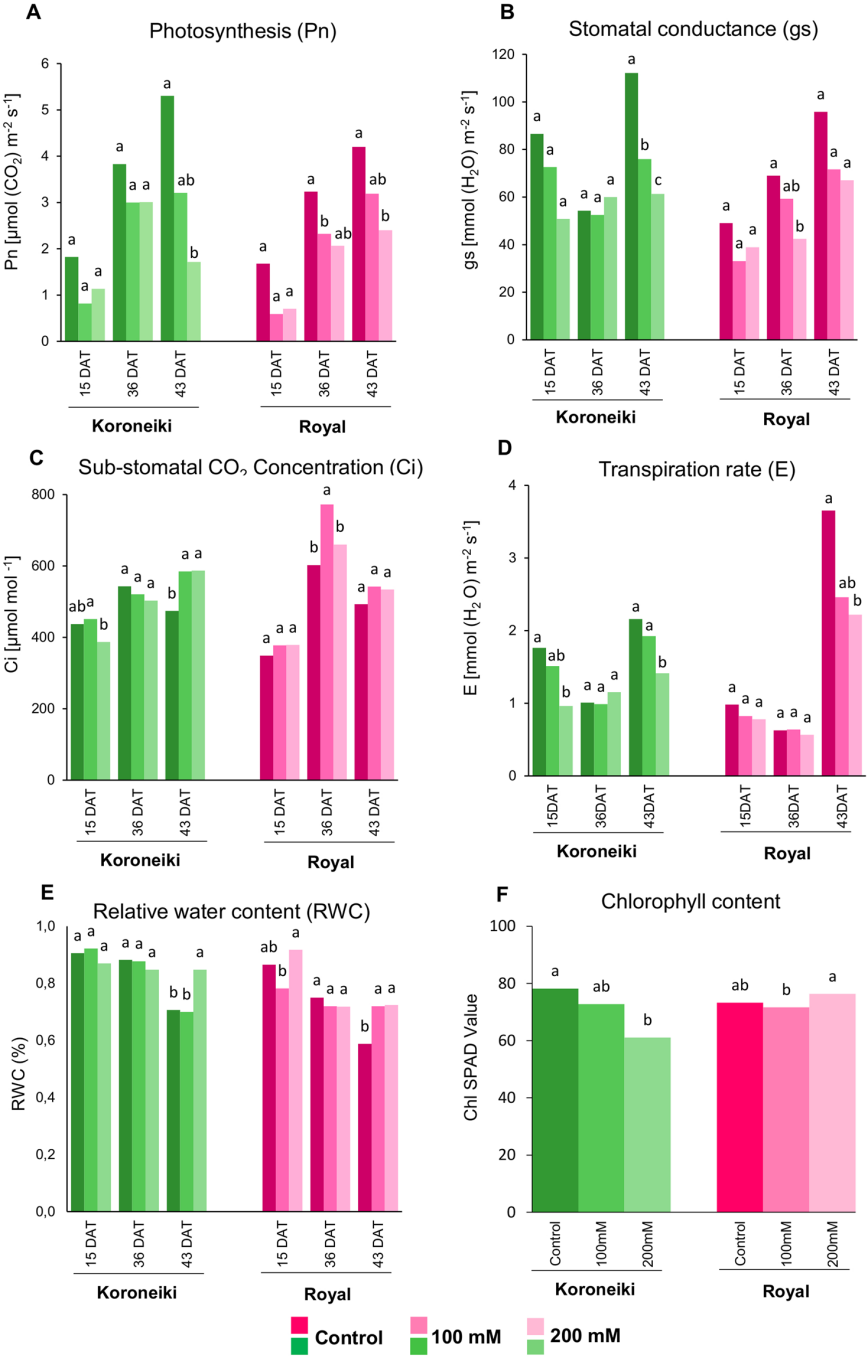
Physiological parameters measured for plants of cultivars Koroneiki and Royal under 0-100-200 mM NaCl at 15, 36 and 43 DAT (except for chlorophyll content, data showed only for 43 DAT) of treatment. (**A**) Net photosynthesis (Pn), (**B**) Stomatal conductance (gs), (**C**) Sub-stomatal CO_2_ concentration (Ci), (**D**) Transpiration rate (**E**), (**E**) Leaf Relative water content (RWC), and (**F**) Chlorophyll content. ANOVA tests were performed inside each cultivar for three treatments (0-100-200 mM NaCl) and separately for each time point. Different letters correspond to significantly different values at P ≤ 0.05.

Under salt treatment photosynthesis decreased significantly at 43 DAT in both cultivars respect to control, especially for ‘Koroneiki’ (Fig. 2A). However, for ‘Royal’ the Pn decreased also at 36 DAT. Stomatal conductance (gs) in stressed plants of ‘Koroneiki’ after 43 DAT was lower than in control plants, while for ‘Royal’ any significant reduction (43 DAT) was observed (Fig. 2B). Sub-stomatal CO_2_ concentration (Ci) under salt stress at 43 DAT in ‘Koroneiki’ increased, whereas in ‘Royal’ did not change, except at 36 DAT under 100 mM when it increased (Fig. 2C). In Koroneiki, at 43 DAT the decrease in gs in stressed plants was accompanied by an increase in Ci. In Royal at 36 DAT, gs decreased in 200 mM of NaCl, without an increase in Ci, but this reduction was not detected at 43 DAT. In both cultivars, at 43 DAT and at 200 mM the transpiration rate decreased in stressed plants respect to control (Fig. 2D).

In general, the relative water content (RWC) from 15 to 36 DAT decreased in ‘Royal’, whereas it did not change in ‘Koroneiki’ (Fig. 2E). At 43 DAT, the RWC in general increased in stressed plants respect to controls. In particular, in ‘Koroneiki’ RWC increased at 200 mM of NaCl, while in ‘Royal’ it increased in all treated plants without differences between salt concentrations.

The chlorophyll content did not show any significant change between the two varieties and different salt concentrations at 15 and 36 DAT (data not shown), unless in ‘Koroneiki’ under 200 mM of NaCl, where it decreased (Fig. 2F).

### Mineral content of leaf, stem and root tissues

Na^+^, K^+^ and Ca^2+^ ions content was measured in leaves, shoots and roots of cultivars Koroneiki and Royal at zero and 200 mM NaCl, at the end of the experiment (60 DAT).

**Figure 3:**
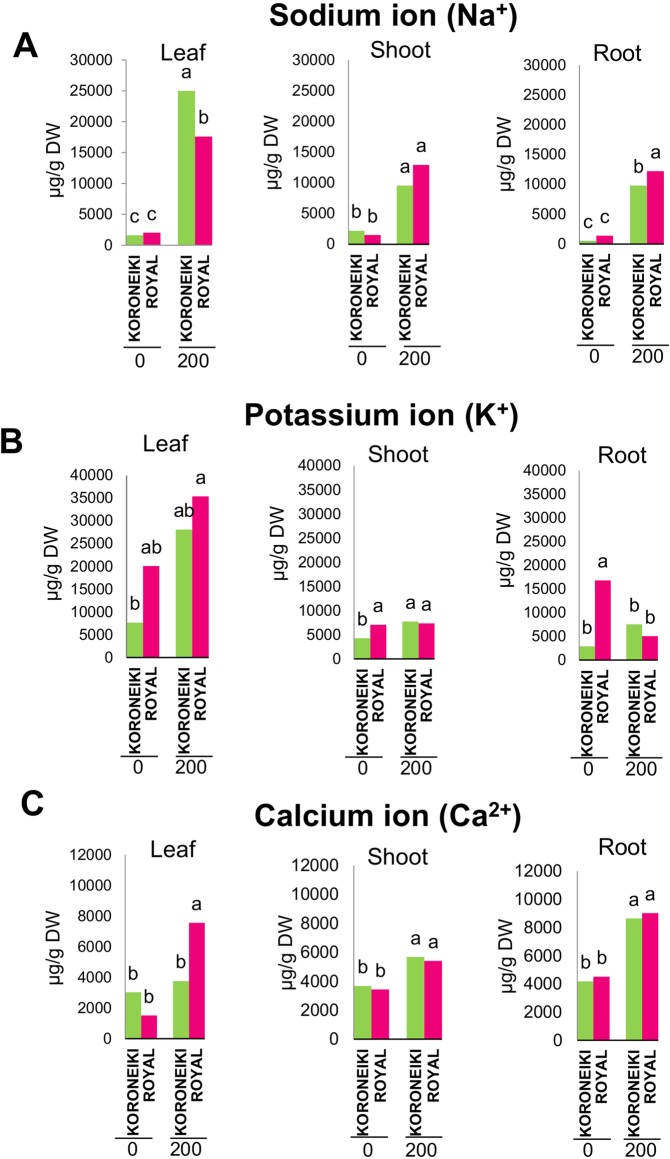
Content of Na^+^, K^+^ and Ca^2+^ ions in leaves, shoots and roots of cv. Koroneiki and Royal plants at zero and 200 mM NaCl, after 60 DAT of treatment. To different letters correspond significantly different values at P ≤ 0.05.

The Na^+^ ion content strongly increased under salt treatment for both cultivars (Fig. 3A). The increase in leaves was higher in ‘Koroneiki’ than ‘Royal’, while in roots, the Na^+^ level was more in ‘Royal’. The K^+^ ion content of leaves increased during the stress in ‘Koroneiki’, while in ‘Royal’ it remained constant.

In shoots, the K^+^ was higher in ‘Royal’, while only in ‘Koroneiki’ slightly increased due to salt stress. In roots K^+^ decreased under salt stress in ‘Royal’, while did not significantly change in ‘Koroneiki’ (Fig. 3B).

Ca^2+^ ion slightly increased under stress in leaves, shoots and roots in both cultivars, especially in ‘Royal’ (Fig. 3C).

### Extent and pattern of DNA methylation under control and salt-stress conditions

Fifteen selective primer combinations (Table S1) were used to generate the MSAP fingerprints from DNA samples isolated from leaves collected at 8, 15, 22, 29, 36 and 43 DAT from cvs. Koroneiki and Royal grown under control and stressed (200 mM NaCl) conditions.

A total of 515 clear and reproducible amplicons were amplified from DNA of ‘Koroneiki’. In control samples, the total methylation of CCGG sequences averaged 58.9%, while in salt-stressed samples it averaged 60.49%. In particular, when compared to control samples, salt stressed plants showed an increase in DNA methylation at all stages with a minimum of 0.4% at 8 DAT to a maximum of 3.1% at 36 DAT (Table 2A).

**Table 2:** DNA methylation changes at 8, 15, 22, 29, 36 and 43 DAT under salinity stress of olive cultivars Koroneiki (A) and Royal (B).

(A) Koroneiki	Control						Salt stressed					
	DAT						DAT					
**MSAP band type (DAT)**	**8**	**15**	**22**	**29**	**36**	**43**	**8**	**15**	**22**	**29**	**36**	**43**
I	200	214	218	206	218	215	198	200	211	202	202	208
II	95	85	75	89	75	85	88	87	77	82	85	90
III	141	133	133	132	133	137	143	140	128	136	138	131
IV	79	83	89	88	89	78	86	88	99	95	90	86
Total amplified bands	515	515	515	515	515	515	515	515	515	515	515	515
Total methylated bands	315	301	297	309	297	300	317	315	304	313	313	307
Fully methylated bands	220	216	222	220	222	215	229	228	227	231	228	217
Hemi-methylated bands	95	85	75	89	75	85	88	87	77	82	85	90
MSAP (%)	61.2	58.4	57.7	60	57.7	58.2	61.5	61.2	59.0	60.8	60.8	59.6
Fully methylated ratio (%)	42.7	41.9	43.1	42.7	43.1	41.7	44.5	44.3	44.1	44.8	44.3	41.1
Hemi-methylated ratio (%)	18.4	16.5	14.6	17.3	14.6	16.5	17.1	16.9	14.9	15.9	16.5	17.5
**(B) Royal**	**Control**						**Salt stressed**					
I	200	204	209	213	213	216	197	199	198	188	190	189
II	77	89	60	65	73	72	81	82	92	88	90	84
III	118	116	95	102	112	111	131	119	117	121	123	120
IV	124	110	155	139	121	120	110	118	112	122	116	126
Total amplified bands	519	519	519	519	519	519	519	519	519	519	519	519
Total methylated bands	319	315	310	306	306	303	322	319	321	331	329	330
Fully methylated bands	242	226	250	241	233	231	241	237	229	243	239	246
Hemi-methylated bands	77	89	60	65	73	72	81	82	92	88	90	84
MSAP (%)	61.5	60.7	59.7	59.0	59.0	58.3	62.0	61.6	61.8	63.8	63.4	63.6
Fully methylated ratio (%)	46.6	43.5	48.2	46.4	44.9	44.5	46.4	45.7	44.1	46.8	46.0	47.4
Hemi-methylated ratio (%)	14.8	17.1	11.6	12.5	14.0	13.9	15.6	17.7	7.0	17.9	16.5	16.2

^a^(II + III + IV); ^b^(III + IV); ^c^MSAP (%) = [(II + III + IV)/(I + II + III + IV)]x100; ^d^Fully methylated ratio (%) = [(III + IV)/(I + II + III + IV)]x100; ^e^Hemi-methylated bands (%) = [(II)/(I + II + III + IV)] × 100. Type I indicated absence of methylation due to the presence of bands in both *Eco*RI/*Hpa*II and *Eco*RI/*Msp*I digest; type II bands appeared only in *Eco*RI/*Hpa*II digestion but not in the *Eco*RI/*Msp*I digest; type III generated bands obtained in *Eco*RI/*Msp*I digest but not in the *Eco*RI/*Hpa*II digest; and type IV represents the absence of band in both enzyme combinations.

A total of 519 clear and reproducible bands were amplified in ‘Royal’. The level of DNA methylation averaged 59.7% in control condition and 62.7% in samples grown under salt stress (Table 2B). The difference in DNA methylation between salt stressed samples and under control conditions increased from 8 DAT to 43 DAT, ranging from 0.6% (8 DAT) to 5.2% (43 DAT). All samples showed a level of full methylation much higher than that of hemi-methylation. Furthermore, differences between stressed and control plants were observed on the type of methylation. In Koroneiki genotypes it was the number of fully methylated fragments to be higher (1.3%) in plants grown under stressing conditions than that revealed in plants grown under control conditions. Conversely, the comparison between stressed and unstressed plants of Royal revealed a higher (2.8%) level of hemi-methylated fragments in the former.

### Effect of salinity stress on the level of methylation in olive

All banding patterns between control and salt stressed samples collected at 8, 15, 22, 29, 36 and 43 DAT were compared for identifying changes in cytosine methylation patterns under salt stress (Table 3). Sixteen banding patterns were originated from the MSAP analysis: patterns A–D represent monomorphic classes in which the methylation pattern did not change in control and salt stressed samples. Patterns E–J indicated cytosine demethylation; whereas patterns K–P showed cytosine methylation events potentially induced by salt stress. Out of 420 and 368 bands (in average), 81.6% and 70.9% of CCGG sites remained unchanged after the regimen of salt stress on Koroneiki and Royal cultivars, respectively. The percentage of demethylated sites under salt stress were 10.1% and the methylated ones were 8.3% in ‘Koroneiki’, whereas in ‘Royal’ values were 13.3% and 15.7%, respectively.

**Table 3:** Analysis of DNA methylation patterns in two olive cultivars under salinity-stress compared to well-fed plants without NaCl (patterns are classified as in Karan *et al*. 2012^18^ and Marconi *et al*. 2013^32^).

	H_2_O			NaCl		Time points					
						cv. Koroneiki					
**Pattern** ^**a**^	**Class**	***Hpa*** **II**	***Msp*** **I**	***Hpa*** **II**	***Msp*** **I**	**8**	**15**	**22**	**29**	**36**	**43**
No Change	A	1	0	1	0	74	65	32	57	59	73
	B	0	1	0	1	127	115	98	108	118	125
	C	1	1	1	1	188	188	197	183	195	195
	D	0	0	0	0	60	56	44	52	58	56
**Total sites**						**449**	**424**	**371**	**400**	**430**	**449**
**%**						**87**.**2**	**82**.**3**	**72**	**77**.**7**	**83**.**5**	**87**.**2**
Demethylation	E	1	0	1	1	1	10	21	6	14	3
	F	0	1	1	1	8	12	10	11	2	2
	G	0	0	1	1	3	4	10	6	7	3
	H	0	1	1	0	1	0	0	2	0	0
	I	0	0	1	0	19	19	30	24	13	14
	J	0	0	0	1	4	9	15	13	12	4
**Total sites**						**36**	**54**	**86**	**62**	**48**	**26**
**%**						**7**.**0**	**10**.**5**	**16**.**7**	**12**.**0**	**9**.**3**	**5**.**0**
Methylation	K	1	1	1	0	1	1	0	6	3	4
	L	1	1	0	1	9	9	11	10	3	0
	M	1	1	0	0	0	2	3	3	1	14
	N	1	0	0	1	1	0	1	1	0	13
	O	1	0	0	0	12	12	23	18	12	5
	P	0	1	0	0	7	13	20	15	18	4
**Total sites**						**30**	**37**	**58**	**53**	**37**	**40**
**%**						**5**.**8**	**7**.**2**	**11**.**3**	**10**.**3**	**7**.**2**	**7**.**8**
						**cv**. **Royal**					
						**8**	**15**	**22**	**29**	**36**	**43**
No Change	A	1	0	1	0	48	50	28	22	50	45
	B	0	1	0	1	88	95	63	82	100	104
	C	1	1	1	1	161	184	151	159	176	169
	D	0	0	0	0	73	76	55	57	78	94
**Total sites**						**370**	**405**	**297**	**320**	**404**	**412**
**%**						**71**.**3**	**78**.**1**	**57**.**2**	**61**.**7**	**77**.**9**	**79**.**4**
Demethylation	E	1	0	1	1	8	9	10	12	6	13
	F	0	1	1	1	16	6	14	13	13	8
	G	0	0	1	1	10	5	24	35	5	7
	H	0	1	1	0	1	5	3	5	2	2
	I	0	0	1	0	19	24	22	19	14	18
	J	0	0	0	1	8	13	11	11	13	11
**Total sites**						**62**	**62**	**84**	**95**	**53**	**59**
**%**						**11**.**9**	**11**.**9**	**16**.**2**	**18**.**3**	**10**.**2**	**11**.**4**
Methylation	K	1	1	1	0	4	10	7	8	11	4
	L	1	1	0	1	28	8	20	8	5	3
	M	1	1	0	0	1	3	20	13	4	23
	N	1	0	0	1	3	0	1	3	3	7
	O	1	0	0	0	25	18	53	51	31	5
	P	0	1	0	0	26	13	37	21	8	6
**Total sites**						**87**	**52**	**138**	**104**	**62**	**48**
**%**						**16**.**8**	**10**	**26**.**6**	**20**	**11**.**9**	**9**.**2**

^a^1. band present at all stages. 0. band absent at all stages.

### Characterizing differentially methylated fragments

From MSAP analysis, 20 polymorphic DMFs among treatments, cultivars and time points were isolated and characterized. After blasting with genomic, transcript and protein databases, six out of 20 DMFs resulted to correspond to the following gene sequences: i) an aquaporin - subfamily membrane intrinsic protein (*OePIP1*.*1*), ii) a cytochrome b6 (*OePetD*), iii) a phosphatidylinositol 4-kinase (*OePI4Kg4*), iv) a xylose isomerase (*OeXylA*), v) a reverse transcriptase within Ty3-gypsy LTR (Long Terminal Repeat) retrotransposon regions (*OeRT-Ty3* gypsy), and vi) an unknown mitochondrial fragment (*OeMit*) (Table 4 and Supplementary Table S2). Full length sequences were obtained in cv. Leccino for three (*OePIP1*.*1*, *OePetD* and *OePI4Kg4*) out of four genes, whereas for the others, only partial and predicted sequences were obtained. Some polymorphisms were detected within the introns of the *OePIP1*.*1* locus and, to a lesser extent, in the exons, presumably corresponding to two different alleles, meanwhile for *OeRT-Ty3* gypsy sequences, the higher level of polymorphisms allowed to infer the presence of three different loci. Specific pairs of primer separately amplified different alleles of loci of these genes. For *OePetD*, *OePI4Kg4*, *OeXylA* and the mitochondrial fragment, no polymorphisms were detected. All sequences were submitted to the NCBI database (Table 4 and Supplementary Table S2).

**Table 4:** Predicted genes and putative biological process of differentially methylated gene fragments.

Predicted gene	Molecular Function	GenBank Accession Number
*Major Intrinsic Protein aquaporin family* (*OePIP1*.*1*)	Ion transmembrane transport. response to water deprivation. water transport	All. A: MF784562 All. B: MF958937
*Cytochrome b6f subunit 4 Chloroplast* (*OePetD*)	Photosynthetic electron transport chain of photosynthesis activity	GU931818.1
*Phosphatidylinositol 4-kinase* (*OePI4Kg4*)	Phosphatidylinositol phosphorylation. Response to salt stress. ATP binding. kinase activity	MF958940
*Xylose isomerase* (*OeXylA*)	Xylose isomerase family protein. carbohydrate metabolic process; located in: endoplasmic reticulum. plasma membrane. vacuole	MG025583
Unknown mitochondrial gene (*OeMit*)	No Gene Ontology terms found	MF958946
Partial putative reverse transcriptase like superfamily. DNA Ty3 gypsy retrotransposon (*OeRT1*)	RNA binding; RNA-directed DNA polymerases. RNA-dependent DNA replication	MF958942
Partial putative reverse transcriptase like superfamily. DNA Ty3 gypsy retrotransposon (*OeRT2*)	RNA binding; RNA-directed DNA polymerases. RNA-dependent DNA replication	AJ536120.1
Partial putative reverse transcriptase like superfamily. DNA Ty3 gypsy retrotransposon (*OeRT3*)	RNA binding; RNA-directed DNA polymerases. RNA-dependent DNA replication	MF958943

The structure of the gene coding for aquaporin plasma membrane intrinsic protein (*OePIP1*.*1*) was characterized by four exons and three introns, including a cds (858 bp) coding for 286 amino acids. Two alleles were found (allele A and B), with a length of 2,476 and 2,464 base pairs, respectively. Differences between alleles included three microsatellite regions (AT, GT and CCCCCA), the first at the beginning of the first intron and the others in 3’UTR, and 34 SNPs, seven in the exons and 27 in the introns and UTRs. Among exon polymorphisms, only one at 652 bp causes an amino acid substitution (Q/K) (Table 4 and Supplementary Table S2). The differentially methylated fragment was located on the third exon of *OePIP1*.*1*. The query for gene ontology terms confirmed the gene function associated to the biological process, cellular component and molecular function already known involved in response to water deprivation (Table 4).

A differentially methylated fragment was found in the fourth subunit of cytochrome b6-f complex (*OePetD*) of the chloroplast genome (olive cv. Frantoio, GU931818.1), involved in photosynthetic electron transport chain. This sequence resulted identical to the Leccino genome and to the transcripts of other varieties. The DMF was placed closed to 5′ end of the gene.

The third gene identified corresponded to a phosphatidylinositol 4-kinase gamma 4-like protein (*OePI4Kg4*). Its structure was characterized by a total length of 5,138 bp, with two exons and one intron, including a cds (1,743 bp) coding for 581 amino acids. The DMF was placed on the first exon. Some polymorphisms were detected between Leccino and Farga cultivars: two microsatellite motifs (a CT repetition at 5’UTR and a polyA in the first intron) and 11 SNPs: seven in the introns and UTR regions and four in the cds, of which one at 1,098 bp causing an amino acid substitution (H/R) (Table 4 and Supplementary Table S2).

Two cloned DMFs corresponded to three reverse transcriptase like superfamily (*OeRT1*, *OeRT2* and *OeRT3*), putatively involved in RNA-dependent DNA replication and RNA-directed DNA methylation. Partial sequences of *OeRT1* and *OeRT3* were 297 and 393 bp, respectively, whereas *OeRT2* corresponded to a sequence previously published in olive as a partial putative gypsy retrotransposon RNase, with 1,024 bp length (AJ536120.1) (Table 4 and Supplementary Table S2).

Another DMF was identified as a xylose isomerase gene (*OeXylA*), involved in carbohydrate metabolic processes. The partial sequence of the gene (1,877 bp) included eight exons and eight introns and the partial cds (633 bp) encodes for 211 amino acids. The DMF was located within the hypothetical seventh intron (Table 4 and Supplementary Table S2).

The last DMF arose from MSAP analysis matched with an unknown mitochondrial sequence (*OeMit*), 779 bp long, coding for a hypothetical protein (Table 4 and Supplementary Table S2).

### Gene expression analysis

**Figure 4:**
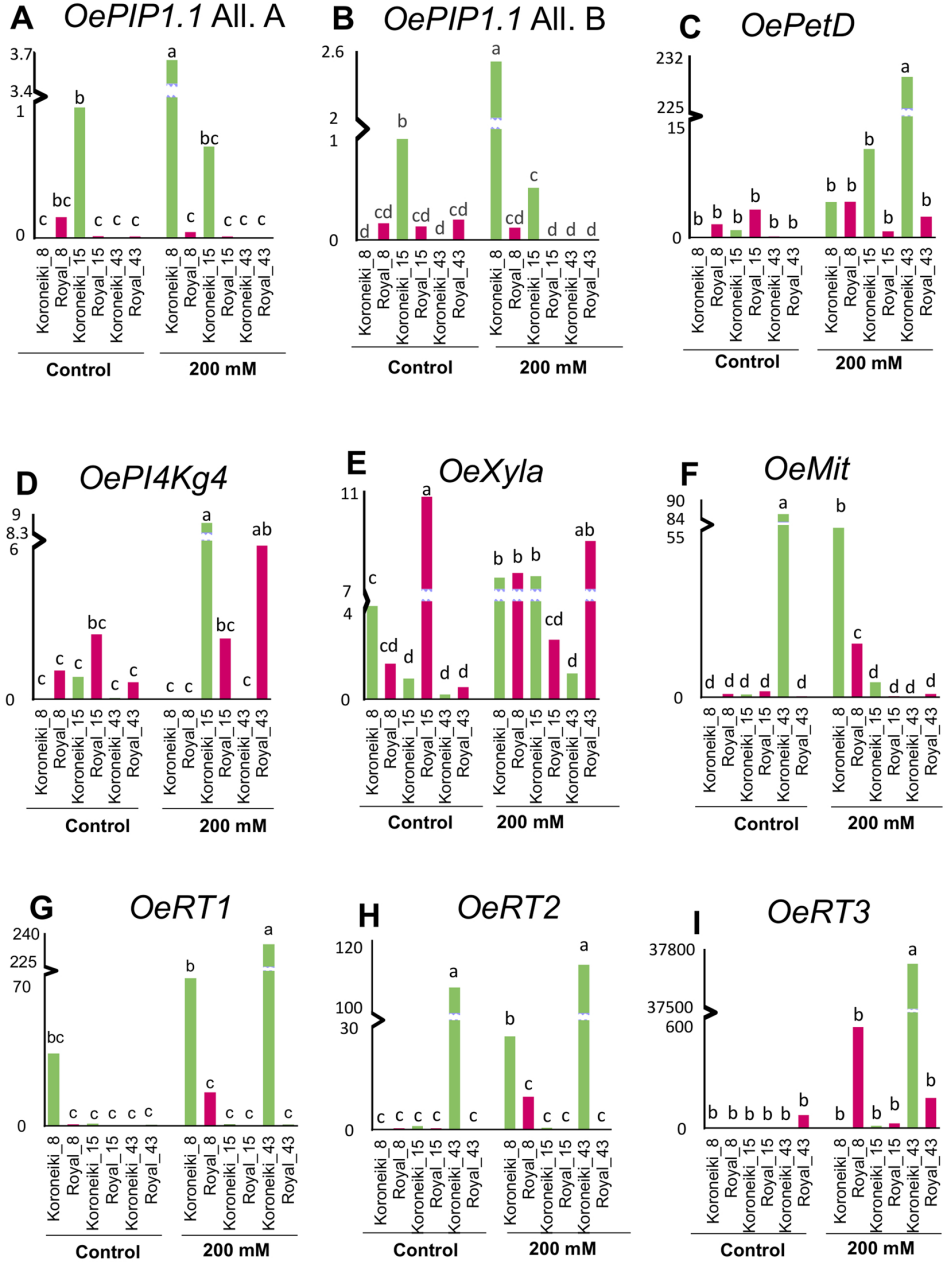
Relative mRNA levels of differentially methylated genes under salt stress, as determined by RT-qPCR. Leaves of plants under control conditions or treated at 200 mM NaCl were analyzed at 8, 15 and 43 DAT of treatment. Transcripts analyzed: (**A,B**) Alleles (**A,B**) of an aquaporin - subfamily membrane intrinsic protein (*OePIP1*.*1*); (**C**) a cytochrome b6 (*OePetD*); (**D**) a phosphatidylinositol 4-kinase (*OePI4Kg4*); (**E**) a xylose isomerase (*OeXylA*); (**F**) an unknown mitochondrial region (*OeMit*); and (**G,H,I**) Three reverse transcriptases Ty3-gypsy (*OeRT1-2-3 Ty3-gypsy*). Values are means of three biological replicates and three technical replicates. To different letters correspond significantly different values at P ≤ 0.01.

The results of qRT-PCR analysis showed different levels of expression between control and salt stressed plants of the two varieties and gave significant results for all analyzed loci (Table S3 and S4). Both alleles of *OePIP1*.*1* showed a similar pattern of expression, with very low levels in treated and control plants of ‘Royal’ at all stages and a clear up-regulation only at beginning of salt treatment in ‘Koroneiki’ (8 DAT), followed by a sharp decrease up to zero at the end of treatment. A narrow difference between allele A and B was observed in Koroneiki cultivar, where the expression level between 8 and 15 DAT was higher for All. A than All. B in stressed plants (Fig. 4A,B).

The plastidial gene, *OePetD* showed an increasing pattern of expression only in stressed plants of the susceptible cultivar and a low expression in all ‘Royal’ control and stressed samples (Fig. 4C).

The *OePI4Kg4* maximum expression was at 15 DAT in stressed susceptible ‘Koroneiki’, while in ‘Royal’ the expression increased during salt treatment, with a prominent peak at 43 DAT (Fig. 4D).

The expression of *OeXylA*, resulted lower in ‘Koroneiki’ than ‘Royal’ plants at 15 and 43 DAT under control conditions. In salt-treated samples the expression was high in both genotypes at beginning of treatment, but at 43 DAT it was maximum for ‘Royal’ and minimum for ‘Koroneiki’ (Fig. 4E).

The unknown mitochondrial gene (*OeMit*) showed a very low expression in all cases, excepting in ‘Koroneiki’ and ‘Royal’ at beginning of stress (Fig. 4F).

*OeRT Ty3-gypsy* 1 and 3 resulted highly expressed only in treated susceptible ‘Koroneiki’ plants, with a dramatic up-regulation at the end of salt treatment, whereas no significant level of expression was observed in any sample of the resistant cultivar (Fig. 4G–I). *OeRT2* was down regulated in both cultivars, even if it was highly expressed during the first days of salt treatment (8 DAT), in both cultivars (Fig. 4H).

The univariate analysis, performed on the unique dependent value (qPCR expression) versus three fixed factors (treatment, genotype and time), showed a strong significant difference (<0.001) for all the correlations with few exceptions. The level of expression of *OePIP1*.*1* All. A and *OeMit* did not depend to salt treatment. Moreover, the ‘genotype’ effect was not significant on the expression of *OePI4Kg4*, while the time was the most significant factor on expression level of all studied genes (Table S4).

## Discussion

In the present study, a multidisciplinary approach was applied to shed light on the physiological, genetic and epigenetic changes activated in olive trees in response to salinity stress. Several researches, based on agronomical^14,47–49^ and physiological data^13,50–53^, showed that olive owns a certain tolerance to salt, but a wide variability of response has been reported, mainly referred to the genetic background of cultivars^54–56^. However, the number of cultivars so far evaluated is very limited if compared to the thousands of genotypes under cultivation worldwide. Therefore, for the vast majority of cultivars, no information on salt tolerance or plant response mechanisms is available.

Previous works, aimed at identifying the genetic determinants of salt tolerance in olive through microarray, next-generation sequencing of transcriptomes or expression analysis of candidate genes, allowed to identify several differentially regulated genes^40,57^. In particular, some transcription factors and transcriptional regulatory networks active against salt stress were identified^9^.

In the present work, the plant growth and stress symptoms, under salt stress and hydroponic system, on five genotypes including one sample of subspecies *cuspidata* were examined. Stress symptoms suddenly appeared in plants of the latter genotype, highlighting its extreme susceptibility to salt, and did not allow its comparison with selected cultivars, while Arbequina and Picual cultivars, showing intermediate symptoms and plant growth. The immediate negative response of subsp. *cuspidata* plants to salt stress was not expected, considering their origin from a strongly harsh environment^106^, probably not including salty soil. It can be assumed that hydroponics itself may have a negative effect on olive plants in direct contact with saline water, regardless of salt concentration to which they have undergone. The cultivar Koroneiki showed a continuous growth of apical leaves under 100 mM of NaCl, while in cv. Royal, the growth of apical leaves stopped under salt treatments. This observation could be related to a system of avoidance under salt stress in ‘Royal’, which is probably not present in ‘Koroneiki’. Considering the different response of the studied genotypes to salt stress, the cultivars Royal, as tolerant, and Koroneiki, as susceptible, were selected for physiological and molecular analyses.

In both cultivars, the decline in Pn of salt stressed leaves was associated with the Ci increase. The increase in Ci due to reduction in Pn causes stomatal closure, with a consequent decrease in gs, in agreement with previous reports^58^. This suggests that the reduction of Pn was caused primarily by non-stomatal effects but could be the result of damage to the photosystem induced by salt stress^59,60^. In particular, similarly to that observed by Proietti *et al*.^61^ in olive senescing leaves, the gs reduction in stressed leaves does not seem to be caused by the loss of guard cell functionality, since the leaf water status did not decrease. In fact, the RWC was not affected in ‘Royal’ and was only slightly decreased in ‘Koroneiki’ by salt treatment. This indicates that leaves were able to maintain cell turgor regardless of hydroponic solution salinity level. Higher Pn, gs and E have been observed in olive cultivars more tolerant also to drought stress^62^.

In both cultivars, Na^+^ concentration under salt stress significantly increased in shoots and dramatically accumulated in leaves and roots. Moreover, the ratio between Na^+^ concentration in roots and leaves in ‘Royal’ was significantly in favour of roots when compared to ‘Koroneiki’, evidencing an ability of the tolerant cultivar to protect above ground meristematic tissues from the accumulations of Na^+^^63^. These results are in agreement with those reported by Kasirga and Demiral (2016)^64^. Furthermore, the K^+^/Na^+^ ratio in ‘Royal’ leaves was higher than in ‘Koroneiki’ after the addition of 200 mM NaCl, confirming salt tolerance as a consequence of both the ability to prevent Na^+^ accumulation and to maintain high levels of potassium in leaves^56,65,66^.

In both Koroneiki and Royal cultivars, the level of DNA methylation increased when plants were subjected to salt stress at all time points. These changes were more evident in salt-tolerant cv. Royal (average differences, 3%) than in the salt-susceptible Koroneiki (average differences, 1.6%)^40^. This indicated higher DNA methylation events in Royal variety than Koroneiki. Furthermore, in Koroneiki cultivar, demethylation was higher in all time points but decreased dramatically (40 sites, 7.8%) at 43 DAT. ‘Koroneiki’ and ‘Royal’ showed a level of full methylation higher than hemi-methylation, as observed in barley plants grown in hydroponic conditions under control and Fe^+^ deficiency conditions^29^. It was notable that differences between treated and control plants were detected between hemi-methylations in the tolerant cultivar and full methylation in the susceptible one.

Through MSAP method, seven DMFs have been detected which were correlated with genes or sequence fragments involved in plant stress response and some of them were directly related to salt stress, such as *PIP1*.*1*, *PetD* and *PI4Kg4*, as reported in previous studies^42,67,68^. These genes were completely predicted and annotated for olive in the present work. All differentially methylated genes showed changes on the level of expression under salt stress, between genotypes and among time points.

Based on factorial analysis, among the studied factors, ‘time’ had the most significant effect on the gene expression. In fact, some genes were over expressed at the beginning of salt treatment, while others were expressed when the salt symptoms were at full-blown. The highest differences of DNA methylation level between control and stressed plants appeared at 15 DAT in ‘Koroneiki’ and at 43 DAT in ‘Royal’, corresponding to the highest level of expression of *OePI4Kg4* at the same DAT, among all studied genes.

Aquaporin gene, belonging to the family of major membrane intrinsic protein (MIP), was demethylated under salt stress in the susceptible cultivar. In plants, MIP family is divided into five subfamilies, including the plasma membrane intrinsic proteins (PIPs)^69^. PIPs are tetrameric complexes with six transmembrane domains. Many PIPs are aquaporins (AQPs), which are involved in water and nutrient permeability^70–72^. PIPs improve Na^+^ exclusion in roots, tissue compartmentalization of Na^+^, water uptake by roots and leaf cell hydration. The up-regulation of AQPs under salt treatment and its relation to the enhancement of photosynthetic activity and stomatal conductance was already reported^69^. In this study, the reduction of Pn and gs in cv. Koroneiki at 43 DAT in stressed plants correspond to the reduction of *OePIP1*.*1* expression in both alleles. Recent studies have shown that the transport of small molecules by PIPs has an important role in defence against biotic and abiotic stresses^73^. Moreover, a strong correlation between gs and the expression of some particular AQPs during water stress in grapevines has been recently reported^74^. As aquaporins accumulate in cells around stomatal cavities and in guard cells themselves^75^, they may also be involved in the regulation of gs. In the present work, two different alleles were found for *OePIP1*.*1*, Allele A perfectly corresponded to the one published by Secchi *et al*. (2007), which was reported as involved in water transport^76,77^ and it was differentially regulated in olive plants under drought stress^78^.

In chloroplast genome, *PetD* (CytB6) is a component of the plastoquinone-plastocyanin reductase^79^, involved in electron transport and generation of ATP^80^. Under salinity stress, CytB6 and other chloroplast protein complexes up-regulated^34,81^. In accordance to what previously reported, in the present work, *OePetD* in the susceptible cv. Koroneiki was increasingly up-regulated, especially at the end of NaCl stress, while, under this condition, photosynthesis and chlorophyll content showed a significant parallel decrease. On the contrary, in cv. Royal, the expression of *OePetD* did not show significant variations under stress conditions, and photosynthesis and chlorophyll under high salt concentration did not change as well. This result agrees with the role of this gene in electron transport^82^.

Phosphatidylinositol 4–kinase (*PI4Kg4*) is a key enzyme in phosphatidylinositol metabolism and leads to the release of Ca^2+^ into the cell cytoplasm^83^. Moreover, PI4Kg4 generates PI4-phosphate, the precursor of regulatory phosphoinositide, involved in many functions including lipid and protein-mediated signalling transduction^84^. *OePI4Kg4* gene may play a regulatory role in increasing Ca^2+^ concentration in all parts of stressed plants, thus limiting the toxic effect of Na^+^ on the integrity of the plasma membrane^85^ and contributing to Na^+^ exclusion^12,64^. Studies carried out in plants under drought or salt stress have shown the up-regulation of *PI4Kg4* gene^68,86^. The up-regulation of *PI4Kg4* gene involved in PI and ABA signalling pathways has been reported in sweet potato plants under salt, drought and nematode stresses^87^. In our experiment, this gene up-regulated in the tolerant Royal cultivar under salt stress, especially at the end of treatment, confirming its role to increase the tolerance to high salt levels (200 mM). The up-regulation of this gene can indicate a mechanism of avoidance, which is playing a role to intensive increase of Ca^++^ in ‘Royal’ roots and limiting the toxic effect of Na^+^ and finally increasing the tolerance of this cultivar after a prolonged exposure to salt stress.

Xylose isomerase (*XylA*) catalyses the conversion of D-xylulose into D-xylose, the primary sugar of the cell wall hemicellulose fraction^88,89^. Previous studies have demonstrated that *XylA*, involved in cell wall modification, accumulates during salt exposure and hemicellulose content of the cell wall increases in salt treated seedlings^90–92^. In our experiment its expression increased in both cultivars under salt, particularly in the resistant Royal cultivar. It may be considered that this gene plays an indirect role in the ability of olive plants to cope with the salt stress, without increasing their tolerance, but regulating growth and dry weight.

The mitochondrial fragment *OeMit*, showed high identity with that of *Boea hygrometrica*^93^, a species well known as drought stress resistant, may be considered as putatively involved also in salt stress by controlling pathways of cell respiration. However, its real function is far to be confirmed and should be deeply evaluated.

The *OeRT Ty3-gypsy* genes belong to the Ty3 gypsy LTR class of retrotransposons, potentially involved in the complex machinery of RNA-directed DNA methylation (RdDM) mechanism^37,94^ and neighbouring genes are usually involved in RNA-directed DNA polymerase and reverse transcription activities^95–98^. Due to sequence variability in the noncoding regions, particularly in the LTR, only a small number of complete LTR retrotransposon sequences have been isolated from plants^99,100^, making difficult further characterization of their function under salt stress. In the present work, *OeRT*1 and 3, were up-regulated in ‘Koroneiki’ and down-regulated in ‘Royal’^101^. The difference in expression in susceptible and tolerant cultivars can show the influence of salt treatment on their expression, while *OeRT2* had the same pattern of expression in both cultivars, which could confirm its down-regulation was not directly related to the salt stress. Some LTR RTs can be activated and transpose as a result of abiotic stress stimuli, including heat, drought and salt stress^102^. In addition, they can also reprogram genes circuits and adjust their performance in response to the specific environmental stimuli^103^.

## Conclusions

In olive plants under severe conditions of salt stress the increase of sodium ions concentration in leaves was higher than in shoots and roots, meanwhile calcium ions increase was limited in all parts, and potassium did not change in roots and shoots but only in unfallen leaves. To this physiological and metabolic scenario, corresponded an impressive appearance of stress symptoms in the susceptible cultivar Koroneiki, started with the reduction of Pn and followed by plant desiccation, whereas plants of the tolerant cv. Royal reduced growth but remained alive along the entire salt treatment period.

Under salt stress conditions, olive plants have undergone important changes in DNA methylation and some of the differentially methylated genes were expressed differently between susceptible and tolerant cultivars under salt stress conditions, confirming their role in plant response to salt stress. In particular, the tolerant cv. Royal de Cazorla responded to salt stress slowing down the expression of four out of six identified genes and therefore its growth, while the susceptible cv. Koroneiki was continued to grow normally until the salt concentration on its tissues brought it to death. This multidisciplinary approach can be applied as innovative methodology to detect and validate genes involved in abiotic stress in plant.

## Materials and Methods

### Plant material, treatments and growing conditions

**Figure 5:**
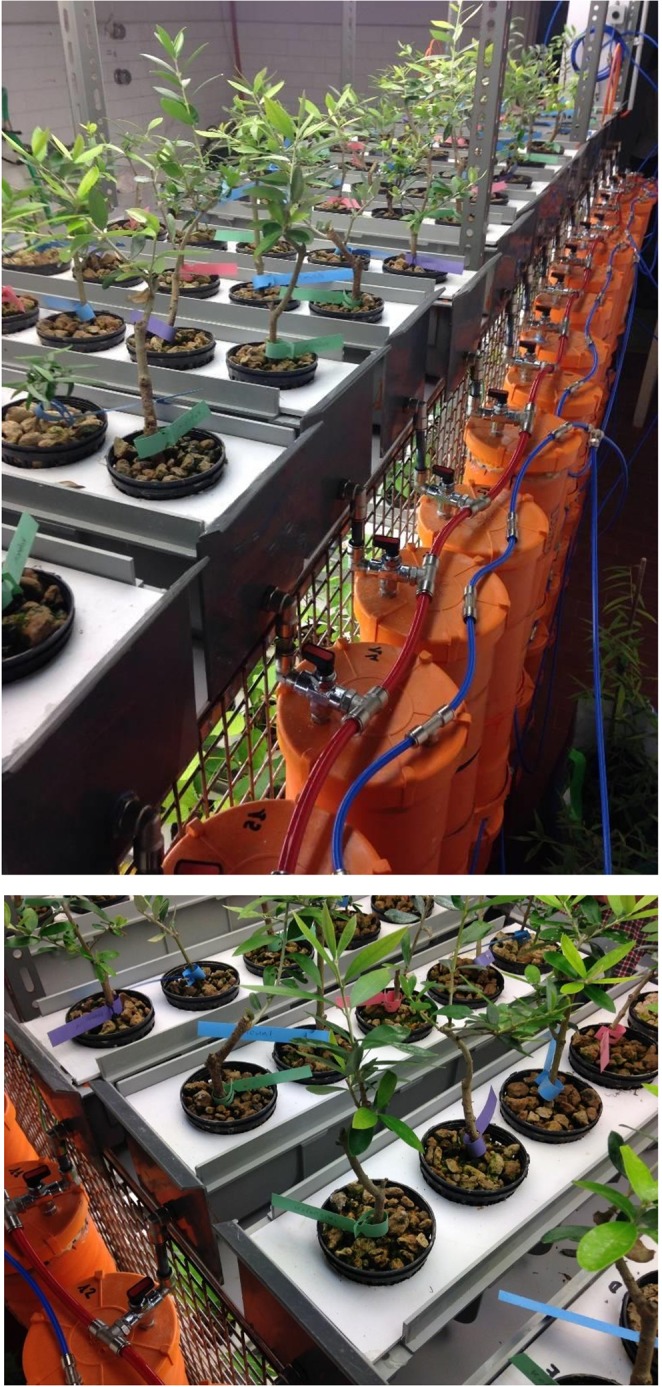
Olive plants of four olive cultivars (Koroneiki, Royal, Picual and Arbequina) and a sample of *O*. *europaea* subsp. *cuspidata* kept in hydroponics under different levels of salt stress (0-100-200 mM NaCl).

Four olive cultivars and a sample of subsp. *cuspidata* (Nepal, Bajura district), with different potential to salt tolerance, were used in this work: cv. Koroneiki, reported as low salt-tolerant^12,104^, Arbequina and Picual, as medium tolerant^105^ and cv. Royal de Cazorla (from now on referred in the text as ‘Royal’) as high-tolerant based on empirical observations (Baldoni, pers. comm.), meanwhile subsp. *cuspidata* was selected as a genotype naturally growing in harsh conditions^106^. Rooted plantlets of these genotypes were propagated by cutting. Plants, with 30 cm of average height, were removed from the perlite and roots were washed with distilled water and placed in 800 ml pots containing expanded clay. Rooted cuttings were grown under hydroponic conditions for an adaptation period of 60 days before salt treatment. The recirculating hydroponic solution was composed of a half strength Hoagland solution at pH 7.5^107^. Control plants were grown in salt-free nutrient medium while treated plants were supplemented with two NaCl concentrations (100 and 200 mM) in the solution. Plants were distributed in randomized blocks in PVC containers, each including plastic hydroponic pots, with five olive genotypes and five replicates/treatment for a total of 15 plants/genotype (Fig. 5). The hydroponic system was located in a growing chamber and plants were exposed to light system equipped with lamps (PHILIPS SON-T AGRO 400 W) producing 200 µmol m^−2^ s^−1^ photon flux density of photosynthetic active radiation, under a photoperiod of 16 h d^−1^. Using an air conditioner, the temperature and relative humidity were maintained at 23 °C (±1.5 °C) and 60% (±3.5%), respectively. The nutrient solution was replaced every week, an automated system ensured the flux of the nutrient solution at different NaCl concentrations three times per day.

### Plant growth and symptoms

During the experiment, the appearance of visible symptoms of stress (e.g. leaf desiccation) was evaluated at regular intervals (8, 15, 22, 29, 36 and 43 days after treatment - DAT) and the eventual occurrence of plant death was monitored as well.

At the end of the experiment, 60 days after starting NaCl treatment, roots, shoots, stems and leaves were separated from three plants for each genotype and treatment, to determine their fresh (FW) and dry weight (DW), measured after oven-drying at 95 °C until constant weight was achieved. Moreover, at 60 DAT, the plant height, number of leaves and leaf area, number and lengths of lateral shoots were measured.

All determinations and molecular analyses were performed only on samples from the most tolerant (Royal) and the most susceptible (Koroneiki) varieties among the five tested genotypes, based on the results obtained from physiological analyses (see Results section).

Measuring photosynthesis, gas exchange, leaf relative water content and chlorophyll content Leaf net photosynthesis (Pn), leaf transpiration rate (E), stomatal conductance (gs), sub-stomatal CO_2_ concentration (Ci) and relative water content (RWC) were determined for each genotype and treatment at 15, 36 and 43 DAT. Leaf gas exchange rates were measured using a portable IRGA (ADC-LCA-3, Analytical Development, Hoddesdon, UK) and a Parkinson-type assimilation chamber. Leaves were enclosed in the chamber and exposed to the same light conditions as in the hydroponic system. The flow rate of air passing through the chamber was kept at 5 cm^3^s^−1^. During gas-exchange measurements, the external CO_2_ concentration was about 375 cm^3^m^−3^ and the air temperature inside the leaf chamber was < 1 °C higher than the hydroponic chamber temperature. Measurements were taken under steady state conditions (after about 30 s). Pn, gs and E were expressed on a leaf-area basis. Pn, gas exchange, chlorophyll content and RWC measurements were done on the expanded leaves under the youngest ones.

RWC was calculated as: RWC (%) = [(FW − DW)/(TW − DW)] × 100^108^. In particular, from a sample of leaf the fresh weight (FW) was determined, followed by flotation in distilled water for four h at room temperature (about 15 °C) under dark conditions. Then, after leaf surface drying with absorbent paper toweling, the turgid weight (TW) was recorded, and discs were subsequently oven-dried to a constant weight at about 85 °C (DW).

The chlorophyll content was measured on three leaves for each treatment with a SPAD-502 Chlorophyll Meter (Minolta Camera Co. Ltd., Japan)^109^.

### Mineral content of leaf, stem and root tissues

In order to measure the content of sodium (Na^+^), potassium (K^+^) and calcium (Ca^2+^) ions, tissue samples of control and 200 mM NaCl treated plants were dried at 65 °C for 48 h, finely ground and extracted with diluted nitric acid. The extracts were analyzed for measuring the concentration of Na^+^, K^+^ and Ca^2+^ using a flame photometer (Digiflame, GDV)^104,110^.

### DNA extraction and analysis of DNA methylation

Sub-apical leaves were collected at 8, 15, 22, 29, 36 and 43 DAT randomly from three out of five plants of the cultivars Koroneiki and Royal grown at the extreme stress conditions (0 and 200 mM NaCl). Total DNA was extracted using the GenElute Plant Genomic DNA Miniprep kit (Sigma-Aldrich).

Methylation Sensitive Amplified Polymorphism (MSAP) technique was applied, following the published procedure^29^. A total of 15 primer combinations was used for selective amplification (Table S5). Each amplified sample was separated on an ABI 3130 XL Genetic Analyzer (Applied Biosystems, Foster City, CA).

The procedure of isolating and screening of polymorphic bands was done as described in previously published paper^29^. In particular, amplified fragments were divided into four types based on the presence or absence of bands. The polymorphic fragments were run on acrylamide gels and silver stained with the aim of isolating and sequencing the selected bands. Twenty interesting polymorphic bands were excised from gels; 6 μl of each diluted sample was used as template for re-amplification by PCR in a 16 μL reaction volume using Type-it Microsatellite PCR Kit (Qiagen). All PCR reactions were carried out with the same primer combinations used in pre-selective amplification step with the following profile: 94 °C for 1 min, 30 cycles of denaturation at 94 °C for 1 min, annealing at 50 °C for 1 min, and extension at 72 °C for 1 min, ending with a 20 min extension step at 72 °C. One μl of the re-amplified DNA was cloned into a pCR4-TOPO vector using the TOPO TA cloning kit for sequencing (Invitrogen). Three plasmid DNAs for each transformation were purified from 5 ml of overnight cultures of Escherichia coli in LB medium using the GenElute Plasmid miniprep kit (Sigma). The sequences of both strands of each plasmid were determined after running sequencing reactions (obtained with BigDye Terminator v3.1 Cycle Sequencing Kit, Life Techologies) on an ABI 3130xl Genetic Analyzer sequencer.

### Identification of genes carrying differentially methylated fragments

The differentially methylated fragments (DMFs) were used as queries to identify the genomic scaffolds in a BLAST search: on the genome sequence of cv. Leccino (http://oleagenome.org/)^111^; on the genome of cv. Farga (http://denovo.cnag.cat/genomes/olive/ Oe5 browser)^112^; on a library of fruit transcripts^113^. Gene sequences and transcripts with the best ID values, E-values and hit scores were identified, followed by a nucleotide BLAST search in NCBI database (https://blast.ncbi.nlm.nih.gov/Blast.cgi) and the alignment with gene orthologs. The BioEdit software (http://www.mbio.ncsu.edu/Bioedit) was used to align gene sequences distinguishing exons and introns. ORFs and corresponding proteins were predicted using the ExPASy translate tool (http://web.expasy.org/translate/) and fgenesh (http://www.softberry.com/berry.phtml). Predicted olive proteins and their putative orthologues were aligned through ClustalW (http://www.mbio.ncsu.edu/Bioedit). Homology to proteins of other plant species was verified by BLASTP in the non-redundant protein sequences database of NCBI. TAIR (https://www.arabidopsis.org/Blast) and UNIPROT (http://www.uniprot.org/blast) were used to search gene ontology terms.

The genomic sequence of each gene was amplified on DNA (25 ng) of cvs. Leccino, Koroneiki and Royal by using LA Taq polymerase (Takara Bio Company), with the following PCR program: 98 °C for three min; 50 cycles of 98 °C for 25 s, 58 °C for 25 s, 68 °C for four min and final extension at 68 °C for 10 min. In order to verify sequence homology, portions of these genes were sequenced using BigDye Terminator v1.1 Cycle Sequencing Kit (Thermo Fisher Scientific). The purified PCR products were sequenced on an ABI PRISM 3130 XL Genetic Analyzer (Applied Biosystems, Foster City, CA). The BioEdit software was used to align the sequenced fragments and to identify the polymorphisms within and between cultivars.

### RNA extraction, cDNA synthesis and gene expression analysis

Total RNA was extracted from leaves of both cultivars under control and stress (200 mM NaCl) conditions collected at 8, 15 and 43 DAT, by using the RNeasy Plant Mini Kit (Qiagen), according to manufacturer’s instructions. To avoid DNA contamination, each sample was treated with DNase I (Ambion) and tested by amplifying the reference gene Elongation Factor 1 alpha (*EF1α*). The concentration of total RNA was assessed using a Nanodrop 2000c spectrophotometer (Thermo Fisher, Schaumburg, USA). Single-strand cDNA was synthesized from 500 ng of total RNA using oligo (dT)18 and SuperScript III Reverse Transcriptase (Thermo Fisher Scientific), according to manufacturer’s instructions. cDNA amplification was evaluated by PCR amplification of the *EF1α* gene.

The expression analysis of DMFs was performed by quantitative PCR on the reverse transcribed cDNA (RT-qPCR). Primers for the RT-qPCR experiments were designed using the program Primer3 version 4.0 (Table S5). Reactions were performed on three biological cDNA samples and three technical replicates for each sample. Each reaction contained 3 μl of diluted cDNA (1:10), 0.3 μm of each primer and 5 μl of SYBR Green Master Mix reagent (Thermo Fisher Scientific) in a final volume of 10 μl. The amplification was performed on an ABI 7300 Real-Time PCR System (Applied Biosystems, Foster City, USA), according to the manufacturer’s instructions, by using the following PCR program: 50 °C for two min and 95 °C for 10 min; 50 cycles of 95 °C for 15 s and 60 °C for 1 min, with a final cycle of 95 °C for 15 s, 58 °C for 1 min and 95 °C for 15 s. After each assay, a dissociation kinetic analysis was performed to verify the specificity of the amplification products. *EF1α* and GAPDH were used as reference genes for sample normalization^114,115^. Relative amounts of each transcript were calculated using the 2−ΔΔCT method^116^.

### Statistical analysis

Physiological and molecular data were analysed using ANOVA (p/0.01 = ** and p/0.05 = *, n = 3). Tukey test was used to compare mean values. The univariate analysis was performed, considering three fixed factors (treatment, genotype and time) by using GNU PSPP Statistical Analysis Software (http://www.gnu.org/software/pspp/get.html).

## Supplementary information

Supplementary data

## Data Availability

All information about protocols, materials and methods described in this manuscript are sufficient to replicate the research. Moreover, we are available to share or give new information to the editor and reviewers, if necessary.
