# Supplementary material for: Physiological, epigenetic and genetic regulation in some olive cultivars under salt stress
Source: Sci Rep. 2019 Jan 31;9:1093. doi: 10.1038/s41598-018-37496-5 (PMC6355907; doi:10.1038/s41598-018-37496-5)
Supplement: Supplementary file 1 — Supplementary data [file 41598_2018_37496_MOESM1_ESM.pdf]

Supplementary data

Physiological, epigenetic and genetic regulation in some olive cultivars under salt stress

Soraya Mousavi[1a#], Luca Regni[1#], Marika Bocchini[1], Roberto Mariotti[2], Nicolò G.M. Cultrera[2], Stefano Mancuso[3], Jalaladdin Googlani[3], Mohammad Reza Chakerolhosseini[4], Consolación Guerrero[5], Emidio Albertini[1+], Luciana Baldoni[2+*], Primo Proietti[1+]

[1]Università degli Studi di Perugia, Dept. Agricultural, Food and Environmental Sciences, Perugia, Italy
[2]CNR - Institute of Biosciences and Bioresources, Perugia, Italy
[3]Università degli Studi di Firenze, Dept. Agrifood Production and Environmental Sciences, Florence, Italy
[4]Ferdowsi University of Mashhad, Dept. Soil Science, Mashhad, Iran
[5]Universidad de Malaga, Dept. Molecular Biology and Biochemistry, Malaga, Spain

[a] Present address: CNR - Institute of Biosciences and Bioresources, Perugia, Italy

[#] These authors contributed equally to this work.

+ These authors contributed equally to this work.

[*] Corresponding Author

E-mail addresses and telephone number:

Soraya Mousavi – soraya.mousavi@ibbr.cnr.it

Luca Regni – regni.luca.agr@gmail.com

Marika Bocchini – marika.bocc87@gmail.com

Roberto Mariotti – roberto.mariotti@ibbr.cnr.it

Nicolò GM Cultrera – niccolo.cultrera@ibbr.cnr.it

Stefano Mancuso – stefano.mancuso@unifi.it

Jalaladdin Googlani - jalal.goglani@yahoo.com

Mohammad Reza Chakerolhosseini – reza.rns2007@gmail.com

Consolación Guerrero - cheloguerreroruiz@gmail.com

Emidio Albertini – emidio.albertini@unipg.it

[*]Luciana Baldoni – luciana.baldoni@ibbr.cnr.it - +39 328 3760912

Primo Proietti – primo.proietti@unipg.it

Supplementary Figure S1; Supplementary Tables S1-S5

FIG. S1. Dry weight (DW) of leaves, shoots, stems and roots of cvs. Koroneiki and Royal plants at 0-100-200 mM NaCl, after 43 DAT of hydroponic culture. Data represent the mean value among three plant replicates per variety.

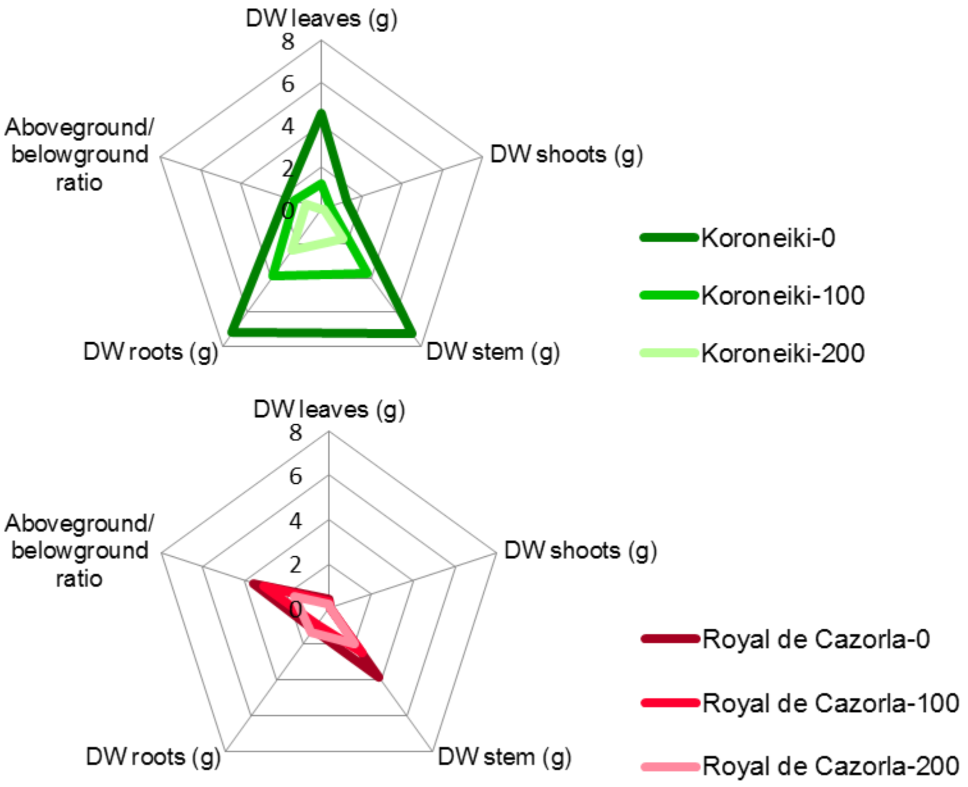

Table S1. Adapter and primer sequences used for MSAP.

**Adapter and Primer**	***EcoR*I (5'-3')**	***Hpa*II / *Msp*I (5'-3')**
Adapter 1	CTCGTAGACTGCGTACC	GATCATGAGTCCTGCT
Adapter 2	AATTGGTACGCAGTCTAC	CGAGCAGGACTCATGA
Preamplification primer	GACTGCGTACCAATTC+A	GATGAGTCTAGAACGG+T
Selective amplification primers	E+AGC<br>E+ACC<br>E+AGA	HM+TGA<br>HM+TCG<br>HM+TAC<br>HM+TGC<br>HM+TCT

Table S2. Annotated genes in *Olea europaea* genome and olive fruit EST libraries.

Predicted gene	Genome Scaffold	Blastn Gene homolog and species	Id%	E-value Hit score	DMF position	Blast	Id %	E-value Hit Score
Major Intrinsic Protein, aquaporin family (*OePIP1.1*)	cv. Leccino 51881.1 All. A<br>cv. Leccino 116227.1 All. B<br>cv. Farga Oe5 s00807 All. A<br>cv. Farga Oe5 s00512 All. B	DQ202708.1 *Olea europaea* plasma membrane intrinsic protein complete mRNA, cds	100	**0.0	Exon 3 - Intron 3	OLEEUCl011899:Contig1<br>OLEEUCl011899:Contig4<br>OE5A055758P1<br>OE5A061883P1	100	**0.0
Cytochrome b6f subunit 4, chloroplast (*OePetD*)	cv. Leccino 450256.1	GU931818.1 *Olea europaea* cultivar Frantoio chloroplast, complete genome	99	**0.0	39-207 bp	OLEEUCl009913:Contig3	100	**0.0
Phosphatidylino sitol 4-kinase (*OePI4Kg4*)	cv. Leccino 20411.1<br>cv. Farga Oe5 s03937	XM_012988396.1 *Erythranthe guttatus* phosphatidylinositol 4-kinase gamma 4, mRNA	100	**0.0	Exon 1	OLEEUCl046382:Contig1<br>OLEEUCl055056:Contig1<br>OE5A052040P1	100	**0.0
Xylose isomerase (*OeXylA*)	cv. Leccino 63314.1 ALL A<br>cv. Farga Oe5 s00118 ALL A	XM_012975859.1, XM_012975858.1 *Erythranthe guttatus* xylose isomerase-like, transcript variant X1, X2, partial mRNA	100<br>99	**0.0<br>**0.0	Intron 7	OLEEUCl018232:Contig1<br>OLEEUCl019977:Contig2<br>OLEEUCl019977:Contig3<br>E8NTSAO01BJNXW<br>E8NTSAO01B8SC1<br>OE5A005580P1	97<br>90<br>92<br>96<br>100<br>92	**e-103<br>**7e-047<br>**3e-034<br>**1e-023<br>**2e-019<br>**7e-051
Unknown mitochondrial gene (*OeMit*)	cv. Leccino 585765.1<br>cv. Leccino 48.1	KX545367.1 *Hesperelaea palmeri* voucher E. Palmer 81 (MO) mitochondrion, complete genome. JN107812.1, *Boea hygrometrica* mitochondrion, complete genome XP_010111288.1, *Morus notabilis* hypothetical protein L484_027941	100<br>100	**7e-159<br>**7e-159	265-396 bp	No hits found	-	**-
Partial putative Reverse Transcriptase like superfamily, DNA Ty3 gypsy	cv. Leccino 757074.1	OTG36861.1 *Helianthus annuus* putative reverse transcriptase domain-containing protein<br>AJ536120.1 *Olea europaea* partial putative gypsy retrotransposon RNase	100	**8e-86	271-406 bp	OE5A057157P1	92	**e-103

retrotransposon (*OeRT1*)		gene, and genes encoding retrotranscriptase and integrase, partial_cds						
Partial putative Reverse Transcriptase like superfamily, DNA Ty3 gypsy retrotransposon (*OeRT2*)	cv. Leccino 177987.1<br>cv. Leccino 239968.1<br>cv. Leccino 229734.1	AJ536120.1 *Olea europaea* partial putative gypsy retrotransposon RNase gene, and genes encoding retrotranscriptase and integrase, partial_cds	93<br>93<br>93	**0.0<br>**0.0<br>**0.0	-	E8NTSAO01BIGJN<br>E8NTSAO01CBCH7<br>OE5A057157P1	94<br>94<br>92	**e-111<br>**e-111<br>**2e-043
Partial putative Reverse Transcriptase like superfamily, DNA Ty3 gypsy retrotransposon (*OeRT3*)	cv. Leccino 327807.1	OTG36035.1 *Helianthus annuus* putative reverse transcriptase domain-containing protein.<br>AJ536120.1 *Olea europaea* partial putative gypsy retrotransposon RNase gene, and genes encoding retrotranscriptase and integrase, partial_cds	100	**2e-173	260-373 bp	OE5A057157P1<br>OE5A066535P1	85	**2e0-56<br>**2e-019

Table S3. One-way analysis of variance on the levels of gene expression (ANOVA) performed on all measurements.

Gene	Mean Square	F	Prob F
OePIP1.1 All. A	Data 3,187<br>Residual 0,050<br>Total 1,036	63,587	5,28E-15**
OePIP1.1 All. B	3,107<br>0,012<br>0,984	269,108	2,42E-22**
OePetD	12,931,290<br>21,264<br>4,078,701	608,125	1,47E-26**
OePI4Kg4	88,999,550<br>1,531,010<br>29,021,120	58,131	1,47E-14**
OeXylA	44,907<br>0,493<br>14,451	91,144	8,25E-17**
OeMit	2,321,845<br>7,245<br>734,690	320,487	3,03E-23**
OeRT1	5,411,069<br>21,667<br>1,715,479	249,743	5,87E-22**
OeRT2	13,918,880<br>166,201<br>4,488,470	83,747	2,2E-16**
OeRT3	3,55E+08<br>6,250,830,000<br>1,16E+08	56,740	1,94E-14**

** Significant at P≤ 0.01.

Table S4. Univariate analysis performed by PSPP software, considering one dependent variable (qPCR results) and three fixed factors (treatment, cultivar and time).

	Source	Type III Sum of Squares	df	Mean Square	F	Sig.
OePIP1.1 All A	Corrected Model	3.58	11	0.33	5.49	**
	Intercept	8.12	1	8.12	136.95	**
	Treatment	0.17	1	0.17	2.83	0.105
	Genotype	1.56	1	1.56	26.27	**
	Time	1.25	2	0.63	10.58	**
	Treatment * Genotype	0	1	0	0.06	0.816
	Treatment * Time	0.2	2	0.1	1.66	0.21
	Genotype * Time	0.4	2	0.2	3.36	0.052
	Treatment * Genotype * Time	0	2	0	0	0.999
	Error	1.42	24	0.06		
	Total	13.12	36			
	Corrected Total	5	35			
OePIP1.1 All B	Corrected Model	34.18	11	3.11	269.11	**
	Intercept	12.91	1	12.91	1117.97	**
	Treatment	0.25	1	0.25	21.3	**
	Genotype	0.09	1	0.09	7.9	*
	Time	11.5	2	5.75	498.01	**
	Treatment * Genotype	6.62	1	6.62	573.08	**
	Treatment * Time	0.06	2	0.03	2.66	0.091
	Genotype * Time	1.28	2	0.64	55.58	**
	Treatment * Genotype * Time	14.38	2	7.19	622.71	**
	Error	0.28	24	0.01		
	Total	47.37	36			
	Corrected Total	34.46	35			
OePetD	Corrected Model	142244.21	11	12931.29	608.13	**
	Intercept	16792.42	1	16792.42	789.7	**
	Treatment	15205.14	1	15205.14	715.06	**
	Genotype	13617.27	1	13617.27	640.38	**
	Time	24071.25	2	12035.63	566	**
	Treatment * Genotype	14583.93	1	14583.93	685.84	**
	Treatment * Time	25306.64	2	12653.32	595.05	**
	Genotype * Time	25190.63	2	12595.31	592.32	**
	Treatment * Genotype * Time	24269.34	2	12134.67	570.66	**
	Error	510.34	24	21.26		
	Total	159546.97	36			
	Corrected Total	142754.55	35			
OePI4Kg4	Corrected Model	252.18	11	22.93	20.15	**
	Intercept	120.28	1	120.28	105.72	**
	Treatment	34.89	1	34.89	30.66	**
	Genotype	1.72	1	1.72	1.51	0.231
	Time	66.83	2	33.42	29.37	**
	Treatment * Genotype	3.94	1	3.94	3.46	0.075
	Treatment * Time	30.96	2	15.48	13.61	**

	Genotype * Time	47.63	2	23.81	20.93	**
	Treatment * Genotype * Time	66.21	2	33.1	29.09	**
	Error	27.31	24	1.14		
	Total	399.77	36			
	Corrected Total	279.49	35			
OeXyla	Corrected Model	493.97	11	44.91	91.14	**
	Intercept	704.06	1	704.06	1428.98	**
	Treatment	79.52	1	79.52	161.4	**
	Genotype	30.12	1	30.12	61.13	**
	Time	54.14	2	27.07	54.94	**
	Treatment * Genotype	6.12	1	6.12	12.42	*
	Treatment * Time	64.95	2	32.47	65.91	**
	Genotype * Time	42.47	2	21.23	43.1	**
	Treatment * Genotype * Time	216.66	2	108.33	219.87	**
	Error	11.82	24	0.49		
	Total	1209.86	36			
	Corrected Total	505.8	35			
OeMit	Corrected Model	25540.29	11	2321.84	320.49	**
	Intercept	7096.21	1	7096.21	979.5	**
	Treatment	50.26	1	50.26	6.94	0.015
	Genotype	3943.18	1	3943.18	544.28	**
	Time	2740.73	2	1370.36	189.15	**
	Treatment * Genotype	503.28	1	503.28	69.47	**
	Treatment * Time	9264	2	4632	639.36	**
	Genotype * Time	2603.19	2	1301.59	179.66	**
	Treatment * Genotype * Time	6435.65	2	3217.83	444.16	**
	Error	173.87	24	7.24		
	Total	32810.37	36			
	Corrected Total	25714.16	35			
OeRT1	Corrected Model	153107.63	11	13918.88	83.75	**
	Intercept	31276.64	1	31276.64	188.19	**
	Treatment	20221.4	1	20221.4	121.67	**
	Genotype	25606.66	1	25606.66	154.07	**
	Time	20737.38	2	10368.69	62.39	**
	Treatment * Genotype	16264.9	1	16264.9	97.86	**
	Treatment * Time	23369.32	2	11684.66	70.3	**
	Genotype * Time	21131.45	2	10565.72	63.57	**
	Treatment * Genotype * Time	25776.53	2	12888.27	77.55	**
	Error	3988.83	24	166.2		
	Total	188373.1	36			
	Corrected Total	157096.46	35			
OeRT2	Corrected Model	59521.76	11	5411.07	249.74	**
	Intercept	16790.1	1	16790.1	774.93	**
	Treatment	391.31	1	391.31	18.06	**
	Genotype	14318.21	1	14318.21	660.84	**
	Time	21192.83	2	10596.42	489.07	**
	Treatment * Genotype	134.38	1	134.38	6.2	0.02

	Treatment * Time	517.61	2	258.8	11.94	**
	Genotype * Time	22857.37	2	11428.68	527.48	**
	Treatment * Genotype * Time	110.05	2	55.02	2.54	0.1
	Error	520	24	21.67		
	Total	76831.85	36			
	Corrected Total	60041.76	35			
OeRT3	Corrected Model	3901371485	11	354670135	56.74	**
	Intercept	370995447.9	1	370995447.9	59.35	**
	Treatment	368253974.2	1	368253974.2	58.91	**
	Genotype	341704105.1	1	341704105.1	54.67	**
	Time	709807000.6	2	354903500.3	56.78	**
	Treatment * Genotype	344277695.4	1	344277695.4	55.08	**
	Treatment * Time	704853030	2	352426515	56.38	**
	Genotype * Time	713710942.2	2	356855471.1	57.09	**
	Treatment * Genotype * Time	718764737.1	2	359382368.6	57.49	**
	Error	150019913.9	24	6250829.74		
	Total	4422386846	36			
	Corrected Total	4051391398	35			

* significant value < 0.01; ** <0.001

Table S5. Specific primers used to amplify methylated fragments for quantitative expression of seven candidate transcripts.

Gene	Primers for RT-qPCR	Nucleotide sequence of primers (5′ – 3′)
OePIP1	qRT- *OePIP1ALL.A*-For	GCTTTTCGAGCCGGGTGAG
	qRT- *OePIP1ALL.A*-Rev	GGCACTTGGAATCAGACTTG
	qRT- *OePIP1ALL.B*-For	ACTTTTCGAGCCGGGTGAG
	qRT- *OePIP1ALL.B*-Rev	TGCACTTGGAATCAGACTTA
OePetD	qRT- *OePetD*-For	AGAGCTAAATTGGCTAAAGGTATGG
	qRT- *OePetD*-Rev	CTACATTACATGCAATAGTACCTAG
OePI4Kg4	*qRT- OePI4Kg4* -For	TCCATACATGGATTTGAATTTGTC
	qRT- OePI4Kg4 -Rev	GCAAGAAAGAGTGGAATACACAAG
OeXylA	qRT- *OeXylA* -For	CACACTATCTAGGTGGAGAAAACTATG
	qRT- *OeXylA* -Rev	ACCATATGATTAAGCTCTCTTTCCAT
OeMit	qRT- *OeMit* -For	CCTTGCTTGAAGCTTGCATT
	qRT- *OeMit* -Rev	TGATTCCTTCCTTGGTTTGG
OeRT	qRT- *OeRT1*-For	GGAGATAATTAGATTAACCATCAAG
	qRT- *OeRT2*-For	GGAGATAAATCGATTAACCATCAAG
	qRT- *OeRT3*-For	AGTTAAAGAAGCAGCTGCAAGAA
	qRT- *OeRT1,2*-Rev	TTGGAAAATACCTTAGCTTCTTTCAG
	qRT- *OeRT3*-Rev	GATGCACATCCTCATGCTTT
